# A perspective of small hydropower in energy transitions in Poland

**DOI:** 10.1038/s41598-025-11149-w

**Published:** 2025-07-14

**Authors:** Maria Dzikuć, Arkadiusz Piwowar, Maciej Dzikuć

**Affiliations:** 1 Faculty of Law and Economics, University of Zielona Góra, Zielona Góra, 65-417 Poland; 2 Faculty of Economics and Finance, Wroclaw University of Economics and Business, Wrocław, 53-345 Poland

**Keywords:** Small hydropower, Foresight, Economy, Renewable energy, Environmental social sciences, Energy science and technology

## Abstract

The article’s subject matter deals with the issue of renewable energy potential and the related issue of energy transition in Poland. The article presents an outline of the issues related to the development of small hydropower plants (SHPs) in Poland against the background of the development of renewable energy. The main purpose of the paper is to demonstrate areas where opportunities and threats are considered for development in this area of RES in Poland. The article uses both secondary and primary sources of information, including the results of empirical research conducted with the Foresight method. The experts indicated, among the others: main problem areas for the development of small hydropower in Poland. The operation of small hydropower plants in Poland does not and will not serve as a basis in the power system. This is mainly due to natural conditions. Nevertheless, it can be an important element in the energy mix, especially from the point of view of energy security at the local level. Small hydropower plants are consistent with the assumptions regarding the development of distributed energy in Poland, including the development of national resources of renewable sources in the energy sector.

## Introduction

In recent years, Poland has been implementing many programs aimed at developing renewable energy. However, despite these activities, the vast majority of electricity, i.e. approximately 70%, is still produced by burning hard coal and lignite. It should be emphasized that the issue of developing renewable energy sources is gaining popularity and importance both in management practices and public debates in Poland and the European Union^1–3^. In the years 2020–2023, there has been a dynamic development of photovoltaics in Poland^4^. Against this background, the development of hydropower is characterized by a relatively low participation rate and poor development dynamics^5^. The share of hydropower in obtaining energy from renewable sources in 2021 in Poland was 1.51%^6^. A number of activities have been undertaken, both in private and public spaces, aimed at increasing the potential of hydropower in Poland. Particularly noticeable activities in this area in Poland include initiatives undertaken by the Polish Water Management Authority^7^. This is an institution established in 2018. The Polish Waters State Water Holding includes the structures of Polish water administration bodies that modernize water infrastructure. In particular, this institution undertakes activities aimed at optimal use of existing hydrotechnical facilities by modernizing or expanding them. Polish Waters also strives to create a comprehensive flood protection system and develop retention activities for agriculture, which allows for the collection of additional amounts of water. This is particularly important in the situation of climate change, which is increasingly affecting Central and Eastern Europe, including drought phenomena^8,9^. The felt effects of drought in Poland concern many areas, including mountain regions^10^. A particularly severe drought in Poland occurred in 2020, which was a consequence of the dry summer of 2018 and 2019, the warm winter of 2019/20 and the very dry spring of 2020^11^.

It should be emphasized that the conditions for the development of hydropower in Poland are influenced by many factors, mainly technical, economic and political ones. Poland is a lowland country with relatively little rainfall. For this reason, the share of hydropower in electricity production in Poland is not significant^12^. Additionally, increasingly frequent periods of drought, especially in recent years, in this part of Europe do not have a positive impact on the development of hydropower^13^. However, there is relatively large potential for the development of hydropower by restoring or modernizing facilities that exist or existed in the past^14^. It is worth noting that in 1935 there were approximately 8,000 hydroelectric power plants and several other installations using hydropower. But currently, the number of hydroelectric power plants in Poland is only about 800^15^.

Hydropower can be a renewable, alternative, flexible option to support large-scale variable renewables^16^. It represents one of the main energy sources for sustainable energy transitions to achieve net zero emissions by 2050^17^. Hydropower can be an important element supporting distributed energy systems and used for the needs of local communities, including smart power grids using renewable energy sources. Recent years in Poland have been a period of dynamic development of small energy-generating installations^18,19^. However, these were mainly photovoltaic micro-installations, of which there were over 1.35 million in Poland in September 2023^20^. The reason for this is geographical conditions (including slight drops in ground level and small and unstable water flows). Therefore, hydropower production in Poland is much lower than the theoretical and technical potential, which is estimated at 15 thousand GWh/year. Unfortunately, currently about 20% of this potential is used. However, despite this, hydropower in Poland can be an important source of renewable energy, the significant advantage of which is the stabilization of the power system^21^. It should be emphasized that Poland is one of the countries that use the potential of small hydropower in the region the least effectively (Table 1). In Eastern European countries, only Belarus and Moldova use the potential of this renewable energy to a lesser extent. It should be added that there is significant diversity in the definition of small hydropower in the region, which makes direct comparisons difficult. The Czech Republic and Romania are leaders in the use of their small hydropower potential. Small hydropower in Eastern Europe still has about 2,289.6 MW of untapped potential (in the ≤ 10 MW category), which is a significant potential opportunity for the development of this renewable energy source in this region of Europe. The development of small hydropower can contribute to increasing the share of RES in the region, but requires a coherent energy policy.

**Table 1:** Small hydropower capacities by country in Eastern Europe (MW)^22^.

Country	Local SHP definition	Installed capacity (local def.)	Potential capacity (local def.)	Installed capacity (≤ 10 MW)	Potential capacity (≤ 10 MW)
Belarus	Up to 10 MW	17.3	250.0	17.3	250.0
Bulgaria	N/A	N/A	N/A	494.7	580.7
Czech Republic	Up to 10 MW	353.0	465.0	353.0	465.0
Hungary	Up to 5 MW	17.1	28.0	17.1	28.0
Moldova	N/A	N/A	N/A	0.3	7.2
Poland	N/A	N/A	N/A	291.7	1,500.0
Romania	Up to 10 MW	321.0	730.0	321.0	730.0
Russia	Up to 30 MW	852.9	825,844.6	168.4	168.4
Slovakia	Up to 10 MW	81.6	145.0	81.6	145.0
Ukraine	Up to 10 MW	119.6	280.0	119.6	280.0
Total	–	–	–	1,864.7	4,154.3

It should be emphasized that small hydropower plants, apart from their main task, i.e. energy production, can also serve as energy storage facilities after certain modifications. The share of this type of energy storage will not have a significant impact on the entire Polish power system, but it could partially solve the problem of surplus electricity generated from photovoltaics^23,24^. This could be particularly important in rural areas dominated by small hydropower plants. Even relatively small energy storage facilities, when used in large numbers, can, at least locally, contribute to reducing the load on the energy system and increasing the use of energy from renewable sources, which cannot always be absorbed by the power grid.

Recent decades have brought a significant increase in demand for electricity in Poland. Despite the implementation of a number of technological solutions in the form of reducing the energy demand of many electrical devices, an increase in the demand for electricity is observed. In the coming years, the demand for energy in Poland is still expected to increase, caused by, among the others, economic growth, digitalization, rising standard of living and the wealth of Poles^25^.

Hydropower plays an important role in energy production based on RES. It should be noted that this is despite periodic declines in the amount of energy produced from this renewable energy source, as was the case in 2018 in Poland due to low rainfall, which was the lowest since 1998. Importantly, significant declines in the amount of energy produced based on hydropower were also recorded in other countries in the region, i.e. Germany, the Czech Republic and Slovakia^26^. The share of hydropower in the EU-27 in total net electricity production in 2020 was 13.8%^27^. This was a much higher share than in Poland, but it should be noted that it is largely inflated by countries such as Sweden, Austria, France and Italy. Analysis of the data presented in Table 2 indicates that hydropower remains an important element of the EU energy mix, although its development has slowed down in recent years. While some countries (e.g. Portugal, Austria) are investing in this type of RES, others (Germany, Sweden) are struggling with the challenges of maintaining existing capacities. It should be emphasized that climate change may be one of the key problems that will affect this type of RES in the coming years. Unstable rainfall and droughts may force a change in the approach to managing existing hydropower resources. In addition, the development of hydropower requires political support and hydropower, especially reservoir hydropower, can act as one of the stabilizers for more unstable sources such as photovoltaics or wind.

The motivation for this study stems from the urgent need to address the pressing challenges associated with sustainable energy transitions and water resource management, particularly in the context of intensifying environmental pressures and evolving policy frameworks. The increasing relevance of hydropower as a renewable energy source necessitates a forward-looking approach to anticipate future developments, assess technological and socio-political dynamics, and inform strategic planning. Accordingly, this study employs a foresight-based methodology to systematically explore potential trajectories and generate actionable insights for decision-makers.

The main purpose of the paper is to demonstrate areas where opportunities and threats are considered for development in this area of RES in Poland. To summarize, the main contributions of this study include: (1) identifying critical barriers and untapped potential in the development of small hydropower plants in Poland, especially in the context of legal and economic frameworks; (2) highlighting the need for a multidimensional assessment approach that integrates technical, environmental, economic, and social factors; and (3) outlining limitations and future research directions, including site-specific hydrological analyses and community engagement strategies necessary for sustainable SHP deployment.

The rest of the paper is organized as follows. The subsequent section presents the *Research Methodology*, detailing the methodological framework and analytical procedures applied. The article uses both secondary and primary sources of information, including the results of empirical research conducted with the Foresight method^28^. The fourth section comprises the *Research Results and Discussion*, wherein empirical findings are systematically analyzed and interpreted in light of the adopted foresight framework. Finally, in *Conclusions and Recommendations*, the concluding remarks of the study are provided by summarizing its key findings and insights.

**Table 2:** Hydropower electricity production capacities in EU countries - in MW^27^.

Specification	2011	2012	2013	2014	2015	2016	2017	2018	2019	2020
European Union—27 countries	142,433.708	143,073.780	144,156.131	144,329.264	146,291.976	147,816.738	148,584.348	148,613.314	148,996.193	148,982.176
Euro area—19 countries (from 2015)	109,781.708	110,455.780	111,299.131	111,931.164	113,438.998	114,784.371	115,393.459	115,471.281	115,831.376	115,891.468
Belgium	1426.000	1427.000	1429.000	1431.000	1422.000	1419.100	1417.100	1417.800	1414.100	1415.800
Bulgaria	3108.000	3181.000	3203.000	3219.000	3219.000	3223.000	3371.550	3379.000	3378.350	3376.456
Czechia	2023.000	2029.000	2064.000	2062.000	2069.000	2071.000	2080.890	2080.598	2080.955	2081.012
Denmark	9.000	9.000	9.000	9.000	6.878	9.267	7.153	7.153	7.263	7.263
Germany	11,367.000	11,185.000	11,197.000	11,190.000	11,212.000	11,164.000	11,078.000	10,652.000	10,698.000	10,757.000
Estonia	5.000	8.000	8.000	5.000	6.000	6.000	7.300	7.300	6.000	8.000
Ireland	237.000	529.000	529.000	529.000	529.000	529.000	529.000	529.000	529.000	529.000
Greece	3224.000	3236.000	3238.000	3389.000	3392.000	3392.000	3392.000	3409.000	3412.000	3417.000
Spain	18,197.000	18,207.000	18,818.000	18,856.000	19,686.000	19,711.000	19,710.000	19,710.572	19,744.667	19,747.592
France	25,454.181	25,469.754	25,458.073	25,398.027	25,368.096	25,435.177	25,517.417	25,542.147	25,674.256	25,496.113
Croatia	2127.000	2127.000	2176.000	2178.100	2192.100	2189.100	2190.300	2196.800	2197.000	2197.200
Italy	21,568.000	21,752.000	21,890.000	21,979.000	22,099.000	22,181.000	22,307.160	22,393.119	22,434.666	22,604.426
Cyprus	0.000	0.000	0.000	0.000	0.000	0.000	0.000	0.000	0.000	0.000
Latvia	1571.000	1573.000	1585.525	1586.748	1586.693	1563.196	1563.260	1563.339	1585.204	1584.755
Lithuania	876.000	876.000	876.000	877.000	877.000	877.000	877.000	877.000	877.000	877.000
Luxembourg	1132.300	1132.300	1132.300	1328.300	1328.300	1328.300	1328.580	1328.508	1328.508	1328.508
Hungary	55.000	56.000	57.000	57.000	57.000	57.000	57.000	57.000	58.000	58.000
Malta	0.000	0.000	0.000	0.000	0.000	0.000	0.000	0.000	0.000	0.000
Netherlands	37.000	37.000	37.000	37.000	37.000	37.000	37.000	37.000	37.000	37.000
Austria	12,642.227	12,773.726	12,848.233	12,997.089	13,112.909	13,570.598	13,717.985	14,088.138	14,162.000	14,169.295
Poland	2345.000	2350.000	2354.000	2363.000	2369.000	2385.000	2389.559	2390.768	2396.512	2399.102
Portugal	5529.000	5706.000	5655.000	5709.000	6162.000	6954.000	7219.731	7229.642	7255.885	7234.706
Romania	6411.000	6455.000	6509.000	6523.000	6619.000	6644.000	6610.437	6617.714	6602.737	6565.675
Slovenia	1137.000	1138.000	1183.000	1180.000	1179.000	1177.000	1230.926	1227.716	1230.090	1230.273
Slovakia	2494.000	2493.000	2493.000	2493.000	2495.000	2493.000	2493.000	2496.000	2494.000	2496.000
Finland	2885.000	2913.000	2922.000	2946.000	2947.000	2947.000	2968.000	2963.000	2949.000	2959.000
Sweden	16,574.000	16,411.000	16,485.000	15,987.000	16,321.000	16,454.000	16,484.000	16,413.000	16,444.000	16,406.000

## Methodology

The methodology of the research conducted in the article is closely related to the assumed purpose of the analyses. During the research number of methods and analyses were used during the research, including:

- analyses based on statistical data,
- mathematical analysis methods,
- tabular and descriptive charts,
- analysis of source documents,
- deductive method and analysis of subject literature,
- Foresight method.

The purpose of forecasting methods is to collect data and give them a useful meaning, so as to enable to look into the future in a different way that has not been considered before. The data necessary for analysis can be collected from people by analysing existing documents and statistical data. The collected data can then be analysed using quantitative and/or qualitative techniques. The information obtained from the analysis and the interpretation of the results can contribute to a better understanding of the past and the present. Thanks to this, the use of the Foresight method can help determine the potential future. The Foresight method allows making more accurate decisions relating to the future strategy. They make it possible to make sense of an uncertain and complex future that depends on many variables. These variables cannot always be precisely determined and/or their intensity of occurrence predicted^29^.

Foresight is a systematic way of obtaining information about the future, which allows defining medium- and long-term development visions. Foresight is a tool that enables better decision-making. The Foresight method was popularized around the world in the last two decades of the 20th century. The aim of research using the Foresight method is to determine future events and needs.

**Fig. 1:**
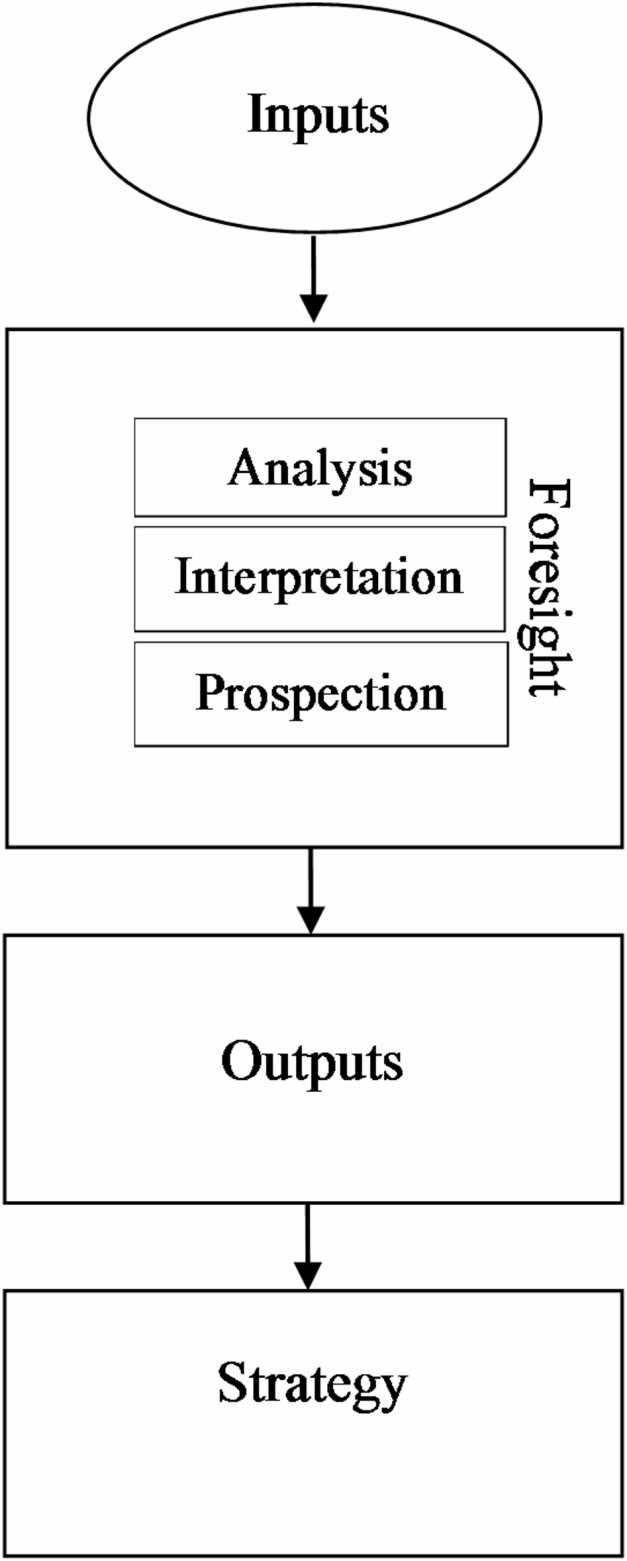
Generic Foresight Model^31^.

This method also makes it possible to identify potential opportunities and threats related to social and economic development. This allows developing and implementing adequate anticipatory actions in a particular field of science and technology. The results obtained using the Foresight method can be used to create and then implement a specific scientific and/or innovation policy in a selected area. The Foresight method assumes the participation of, among the others, representatives of science, public authorities, industry and non-governmental organizations in research^30^. The results of research conducted using the Foresight method can inform decision-makers about probable development trends, the knowledge of which can contribute to making better decisions and creating strategies. Voros’s^31^ foresight process was applied which comports inputs, analysis, interpretation, prospection, and outputs (Fig. 1).

The collected data allowed analysing the development potential of small hydropower plants in Poland. The analyses carried out in the article made it possible to indicate the development potential of small hydropower in the coming years in the context of building the energy mix, taking into account local problems. The research used, among other things, secondary data, i.e. statistical data, information related to the operation and development of small hydropower in Poland and obtained from central and local government authorities.

During the research, respondents indicated the most important problems and potential ways to solve them. In July 2024, research was carried out using the Foresight method. The people who took part in the research included scientists, representatives of energy sector companies, members of non-governmental organizations and local government authorities. The results of the conducted research allow us to indicate the directions of development of the Polish power industry, with particular emphasis on small hydropower plants. The Foresight method allowed for the identification of key areas for the development of the electricity sector, with particular emphasis on RES, including small hydropower plants. The study involved 30 independent experts who have extensive theoretical and substantive knowledge. The group of independent experts studied included scientists, representatives of non-governmental organizations, managers of energy companies (including those managing hydropower plants) and representatives of public authorities dealing with the subject of renewable energy sources. Such a selected group of experts made it possible to obtain the data necessary for analysis and to look at the problem analyzed in the manuscript from a broader perspective. It is worth noting that the experts who took part in the Foresight research are mainly practitioners who have extensive experience in the scope of the problem analyzed in this article. During the analyses carried out using the Foresight method, the desired directions of changes were determined in order to effectively implement the analysed RES into the energy system.

## Problems of the Polish power industry and the potential to eliminate them using small hydroelectric power plants

Many years of neglect in adapting the Polish electricity industry to EU standards have resulted in fossil fuels, including hard coal and lignite, still being the most important sources of energy. The Polish electricity system is currently struggling with many problems. The most important of them include:

- excessive share of coal (approx. 70%) in the total energy balance,
- obsolete electricity transmission lines,
- lack of stabilizers in the form of e.g. energy storage facilities allowing full use of energy generated from RES, e.g. photovoltaics,
- pressure from the EU to increase the dynamics of energy production based on renewable energy sources,
- rising energy prices,
- frequently changing legal regulations, making it difficult for entrepreneurs to plan investments in a long-term perspective.

Some of these problems can be partially solved by the development of small hydropower plants. Even though the natural conditions prevailing in Poland significantly limit the potential of using hydropower, solving even a small part of the problems using RES should be subject to detailed analyses^32^.

It should be emphasized that the potential of hydropower in Poland can lead to a reduction in the share of coal in the total energy balance only to a limited extent. However, a large number of small hydropower plants and other renewable energy sources in Poland may lead to a partial reduction of the existing, mostly outdated energy transmission networks. According to data from World Small Hydropower Development over 80% of the technical production potential of small hydropower plants in Poland is unused mainly due to legal and economic conditions. A systematic departure from the solution based on generating most of the electricity in Poland in a dozen or so large coal-fired power plants may lead not only to a reduction in the use of extensive transmission networks, but also to a positive impact on the increase in the stability of the entire energy system by limiting the role of the largest coal-fired power plants in favour of many distributed sources, based on RES, including small hydropower plants.

In January 2022, there were 785 hydropower plants operating in Poland, the vast majority of which (770 power plants) should be classified as small hydropower plants because their installed capacity was lower than 5 MW (Table 3). Despite the significant numerical advantage of small hydropower plants, their total installed capacity was less than 260 MW (URE, 2024), while medium-sized and larger hydropower plants (i.e. with a capacity above 5 MW) had an installed capacity almost three times higher (over 720 MW).

**Table 3:** Number and installed capacity of hydropower plants in Poland in 2022^33^.

Number of hydropower plants	Size	Power in MW
600	≤ 0.3 MW	44.4
105	> 0.3–1 MW	63
65	> 1–5 MW	152.1
5	> 5–10 MW	36.2
10	> 10 MW	684.4
785	Total	980.1

Recent years have brought asymmetrical development of the Polish renewable energy sector. Favourable legal solutions led to the dynamic development of photovoltaics in 2019–2023. Within a few years, approximately 1.5 million photovoltaic installations were built. However, power grids were designed with one-sided transmission of electricity to final consumers in mind. Most power grids were not designed for bilateral energy transmission at the design stage, which sometimes prevents the energy produced by prosumers from being transferred to the energy system. Especially when a large number of prosumers are located in a small area. Another very serious problem is the too slow development of solutions allowing for the stable development of energy based on RES, which has not been supported, for example, by an increased number of energy storage facilities. Therefore, excess energy produced by photovoltaic installations could not be used or stored in Poland^34^. This led to the need to export electricity outside Poland for an additional fee. This mainly concerned spring and summer sunny days, which were non-working days and it was not possible to use the excess electricity at the time of its production.

**Fig. 2:**
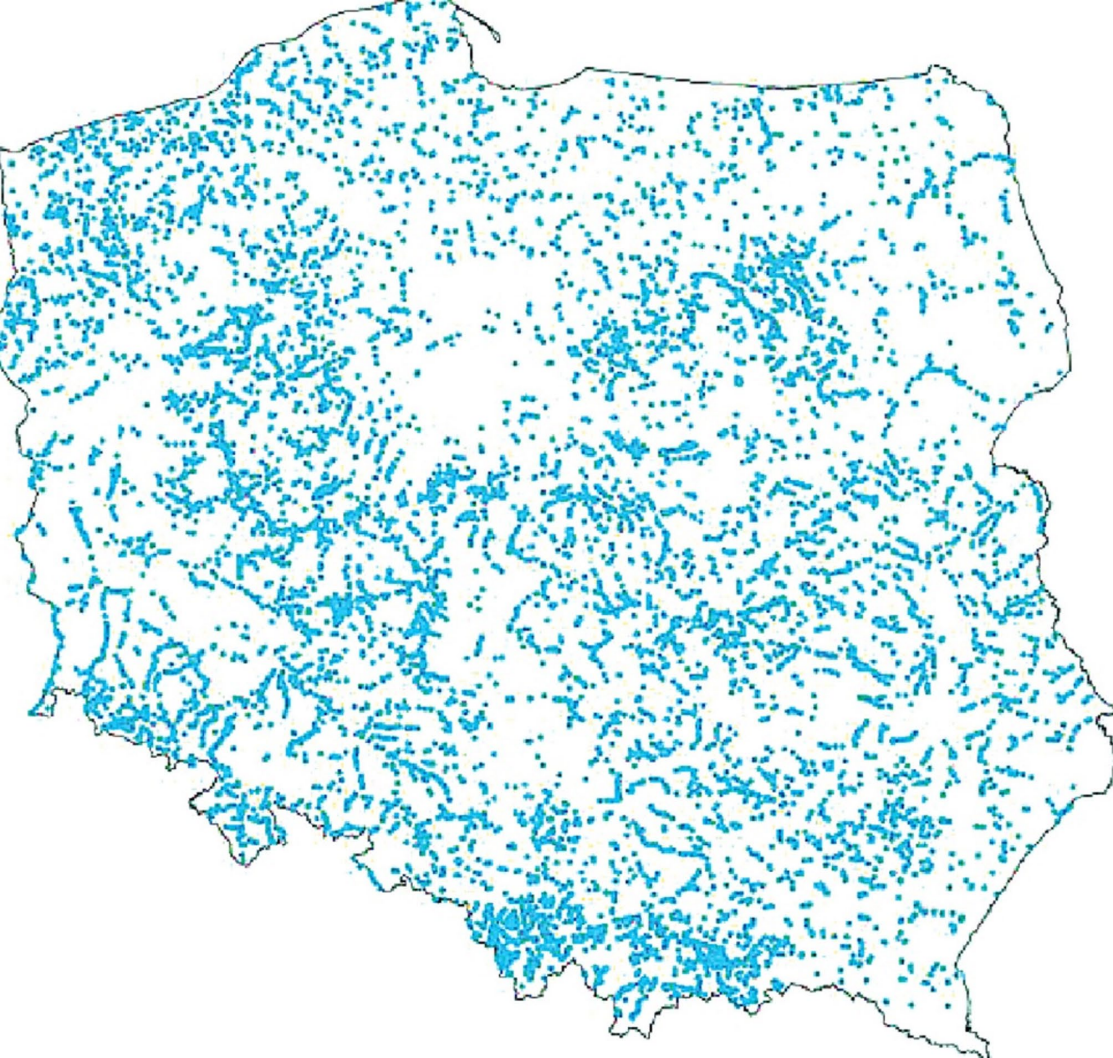
Places for the construction of small hydropower plants^35^.

It should be noticed that small hydropower plants, especially those that have the ability to pump part of the water into the upper reservoir when we are dealing with excess electricity generated from RES, could have partially solved this problem and stabilized the energy system. Although the potential for energy storage using small hydropower plants is limited and a complete solution to the problem requires the simultaneous implementation of various solutions, including the construction of energy storage facilities, the dispersed potential for the construction and use of small hydropower plants in Poland means that at least some of the most attractive locations should consider their implementation (Fig. 2).

**Fig. 3:**
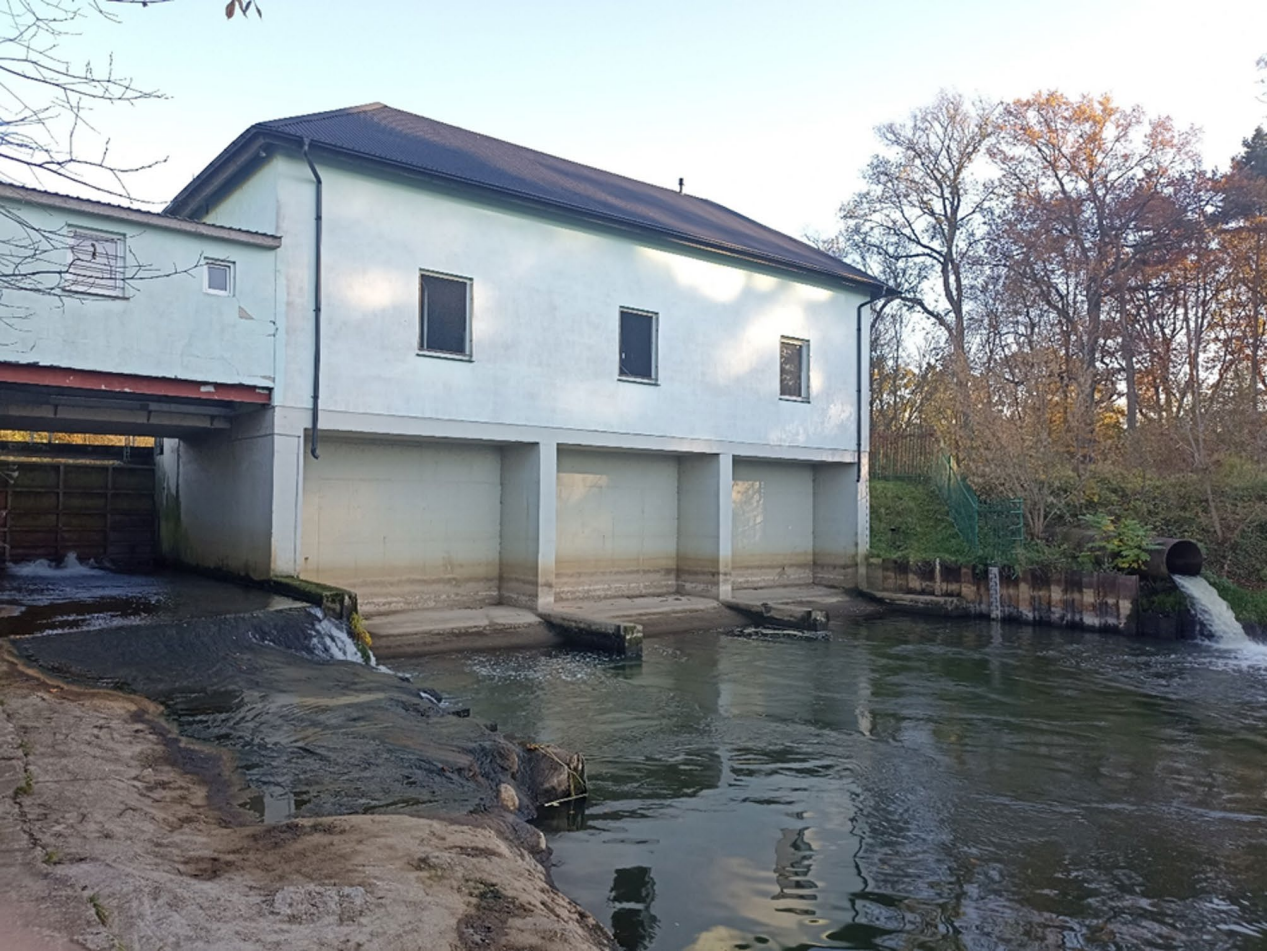
Hydroelectric power plant in Bukowina Bobrzańska (photo by M. Dzikuć, September 2024).

An important advantage of small hydropower plants is the possibility of installing them on smaller rivers or streams, which allows for energy production at a local level. Taking into account the natural conditions occurring in Poland, it is necessary to implement technological solutions that will enable the use of even small river declines in watercourses carrying small amounts of water. An example of a relatively new hydroelectric power plant in Poland is the small hydroelectric power plant “Bukowina Bobrzańska” on the Bóbr River (Fig. 3). Kaplan-1550 turbines were installed in this power plant.

## Research results and discussion

Independent experts with extensive substantive knowledge and experience in the energy sector, including renewable energy, participated in the research. An important element that determined the use of the Foresight method was the fact that the research results can contribute to an effective review and indication of potential directions of development of the Polish electricity sector, with particular emphasis on small hydropower plants. Respondents answering the questions were asked to justify their opinions. The aim of the research, which was conducted in stages, was to achieve consensus among respondents or to obtain a clearer difference in expert opinions. The Foresight method used during the research was aimed at obtaining experts’ views on the likely development of small hydropower plants, which are an element of the Polish electricity sector.

The results of the conducted research demonstrate a number of barriers to the further development of small hydropower plants in Poland. The most important of them include: lack of sufficient financial and legal support for small hydropower plants, complicated and frequently changing law, complicated procedures related to obtaining permits for this type of investment, changing climate and the related repeated and longer-lasting droughts (Table 4). Experts pointed out that the development of small hydropower plants requires stable conditions of regulatory and financial support for potential investors, which has so far been insufficient in Poland, hence renewable energy has hardly been developed at all. Experts have identified a number of solutions that can help in the stable development of small hydropower plants in Poland. These include: enabling financing of this type of investment on preferential terms, which will take into account the specificity of this RES, which in many cases is characterized by a much longer payback period than other RES, e.g. photovoltaics. To ensure long-term viability, strategic planning efforts should embed SHPs within broader rural development agendas, integrated watershed management policies, and circular economy frameworks. Such cross-sectoral alignment would not only enhance the attractiveness of SHP projects for investors but also reinforce their role in advancing Poland’s goals for sustainable energy transition and regional development.

Poland’s principal river systems—including the Vistula, Dunajec, San, Bug, Oder, Bóbr, and Warta—represent the foundational hydrological assets underpinning the country’s technical potential for hydropower development. These rivers are characterized by substantial flow capacity, extensive catchment areas, and existing hydraulic infrastructure, such as weirs and barrages, which collectively enhance their suitability for both large-scale and small-scale hydropower applications.

From a strategic foresight perspective, the concentration of technical potential within these basins presents significant opportunities for targeted investment and policy intervention. In particular, the rehabilitation of disused infrastructure (e.g., historical mill sites, inactive barrages) coupled with regulatory streamlining could substantially expand the distributed generation capacity of the national energy system. Moreover, integrating small hydropower development into broader regional water management frameworks—particularly in the domains of flood mitigation, drought resilience, and agricultural irrigation planning—offers the prospect of multi-dimensional co-benefits. These include enhanced local energy security, improved hydrological balance, and support for the sustainable development of rural and semi-urban areas.

Moreover, experts demonstrated the need to launch a stable system of feed-in tariffs, which will allow investors to properly calculate the investment, which in the next several decades will involve lower maintenance costs of this type of installation than in the case of other RES. In addition to activities aimed directly at potential investors, experts also pointed out the need to carry out work on the drainage and regulation of rivers. According to experts, it is also necessary to look for solutions that will compensate investors for unfavourable climate changes, e.g. reducing river flows. As this type of support, experts indicated a reduction in tax burdens, including local taxes on buildings and the amount of rent for water damming facilities (Table 5). Moreover, experts pointed out that newly built or modernized small hydropower plants must include solutions that limit the impact of the installation on the environment, e.g. facilitating fish migration. Scientific literature often highlights environmental problems related to the development of hydropower plants, including morphological changes in habitats, degradation or fragmentation of aquatic ecosystems^36,37^. These risks need to be minimized and the benefits and risks of hydropower projects carefully assessed, especially in the face of climate change^38^.

**Table 4:** Barriers to the development of small hydropower in Poland.

Specification	Number of responses
Lack of sufficient financial and legal support for small hydropower plants	27
Complicated and frequently changing law, complicated procedures related to obtaining permits for this type of investment	25
Changing climate and the related repeated and longer-lasting droughts	24

As a benefit not directly related to the production of electricity based on the analysed RES, experts pointed out that small hydroelectric power plants can reduce local flooding resulting from intense rainfall. It should be emphasized that, according to experts, an important element of the development of small hydropower plants may be their use as energy storage facilities that will support other renewable energy sources, the production of which depends on changing weather conditions and sunlight. Greater use of small hydropower plants as energy storage will lead to increased flexibility of RES. As mentioned earlier, such a system can act as a bridge between the main sectors of the energy system in Poland. However, adapting small hydropower plants so that they can also function as energy storage requires significant investment outlays related to their reconstruction. Investors will not be able to finance the construction of this type of installation without state support. On the other hand, the literature also adopts a hybridization strategy in which a hydropower unit is connected to an energy storage system (ESS) to increase operational flexibility and mitigate damage to the hydropower plant^39^. Such actions can not only extend the life of the power plant, but also maximize profitability in the event of significant fluctuations in the flow in the watercourse.

**Table 5:** Activities determining the development of small hydropower in Poland.

Specification	Number of responses
Increased financing of small hydropower plants on preferential terms, which will take into account the specificity of this RES	26
Integration of the development of small hydropower with broader regional water management frameworks (including flood mitigation, drought resistance)	23
Increased intensity of land improvement works, with particular emphasis on river regulation	22
Implementation of solutions that will compensate investors for unfavourable climate change	19
Reduction of tax burdens	18

The changing situation on the Polish, European and global energy markets may in the future result in solutions based on small hydropower plants becoming more economically competitive and RES will not require such large financial support from the state. Moreover, as is the case in wind energy or photovoltaics, the issue of disposal and recycling costs of devices should be taken into account. This problem will concern the perspective of 15–25 years. It is necessary to take appropriate actions that will contribute to the optimization of the circular economy in photovoltaics and other RES, in overall sustainable development at the level of production, distribution and use^40^. It is also necessary to implement appropriate waste management action plans^41^, including the design of waste management centres^42,43^. Conventional energy resources, which are depleting, are systematically becoming more expensive over the long term, which leads to constant changes in the economic conditions of energy production based on various energy sources, including renewable ones. It should be emphasized that, additionally, renewable energy sources are not subject to various types of fees, e.g. for CO_2_ emissions, which also affect the changing economic conditions of electricity production depending on the energy source or raw material used^44^.

An important advantage of small hydropower plants is the possibility of installing them even on watercourses with a relatively low slope and low flow. However, the construction or modernization of a small hydropower plant still requires obtaining a number of administrative permits. Although this installation does not emit greenhouse gases, it does affect the environment. Polish law recognizes any land with flowing water as state property, which makes it necessary to obtain the rights to dispose of State Treasury real estate. They can be obtained from the Polish Water Management Authority. It should be emphasized that in Polish conditions this is a long-term process. However, small hydropower technology may gain traction in the Polish energy and power ecosystem owing to incentives and reforms aimed at increasing energy mix diversification for sustainable development. It may be necessary to attenuate some environmental regulations that significantly impact the feasibility of new hydropower investments^45^.

## Conclusions and recommendations

Poland’s strategic objective of achieving a minimum 32% share of renewable energy by 2030 necessitates a decisive shift away from coal-based electricity generation. Within this broader energy transition, the development of small hydropower plants represents a viable and environmentally sustainable alternative, offering both infrastructural adaptability and decarbonization potential. The findings of this study, grounded in a foresight-based analytical approach, underscore the imperative for targeted regulatory interventions and supportive policy mechanisms. These are essential to unlocking the untapped capacity of small-scale hydropower and advancing the national agenda for a low-emission, diversified energy system.

It is estimated that over 80% of the technical production potential of small hydropower plants in Poland is unused mainly due to legal and economic conditions. The current economic and legislative support for small hydropower plants in Poland is too weak an incentive for investors to make many of them consider choosing this RES as a favourable business option. This situation means that not only are new small hydropower plants rarely built, but also many old facilities that can be modernized and used continue to degrade. Activation of appropriate support mechanisms could lead to the restoration of at least some of these types of facilities. Furthermore, current problems related to the use of excess energy from renewable energy sources could improve the profitability of using this type of installations that could act as energy storage facilities. The use of small hydropower plants as energy storage in local conditions has potential, but requires taking into account a number of aspects, including: technical, environmental, economic and social. Moreover, the development of other energy storage technologies may affect the competitiveness of the use of small hydropower plants. The analysis preceding the installation of a small hydropower plant requires a comprehensive approach that will take into account various aspects and interests of the parties that will be involved in the implementation of the project. The modernization, construction and operation of small hydropower plants must take place with full consideration of environmental and social aspects.

From a foresight perspective, the continued development of small hydropower plants (SHPs) should be viewed not only in the context of energy transition, but also as an integral element of rural development policy. Aligning SHP-related initiatives with national and EU-level rural development programs—such as the National Rural Development Strategy or instruments under the Common Agricultural Policy—can generate valuable synergies. These include strengthening local energy self-sufficiency, enhancing regional value chains, diversifying income sources for rural communities, and improving the technical condition of hydrotechnical infrastructure.

Incorporating SHPs into integrated local development plans, however, requires adequate support mechanisms—financial, legislative, and institutional—along with active involvement of stakeholders at local, regional, and national levels. Only through such coordinated action can the full potential of SHPs be realized as tools of both energy transformation and sustainable rural development.

It should be noticed that the analyses conducted in this article have certain limitations. Due to the large dispersion of potential locations for small hydropower plants, it was impossible to conduct a detailed analysis of all installations, which may number in thousands across the country. To precisely determine the potential of individual installations at the local level, further research is necessary, including: average flows of individual watercourses, especially in summer periods when droughts periodically occur in Poland. It is also necessary to determine the detailed environmental impact of small hydropower plants planned to be built or modernized. Moreover, due to the development of single-family housing in rural areas, it would be necessary to conduct public consultations at the local level, because a lot of buildings and other infrastructure have been built around the potential locations of small hydropower plants.

## Data Availability

The datasets analysed during the current study are available from the corresponding author on reasonable request.
